# Selected Blood Inflammatory and Metabolic Parameters Predicted Successive Bilateral Sudden Sensorineural Hearing Loss

**DOI:** 10.1155/2019/7165257

**Published:** 2019-07-08

**Authors:** Xueyuan Zhang, Yinlun Weng, Yaodong Xu, Hao Xiong, Maojin Liang, Yiqing Zheng, Yongkang Ou

**Affiliations:** ^1^Department of Otolaryngology, Sun Yat-sen Memorial Hospital, Sun Yat-sen University, Guangzhou, China; ^2^Institute of Hearing and Speech-Language Science, Sun Yat-sen University, Guangzhou, China; ^3^Department of Neurosurgery, Sun Yat-sen Memorial Hospital, Sun Yat-sen University, Guangzhou, China

## Abstract

**Objectives:**

To explore whether peripheral inflammatory, metabolic, and hemostatic parameters could predict the pathogenesis of successive bilateral sudden sensorineural hearing loss (SSNHL).

**Methods:**

This study reviewed 33 patients with successive bilateral SSNHL and 215 patients with unilateral SSNHL. Clinical characteristics and hematological parameters were compared, including the inflammatory markers (like neutrophil lymphocyte ratio (NLR), monocyte lymphocyte ratio (MLR), and platelet lymphocyte ratio (PLR)) and metabolic features (including hypertension, triglyceridemia, dyslipidemia, and hyperglycemia), as well as hemostatic indices (including prothrombin time (PT), activated partial thromboplastin time (APTT), and fibrinogen).

**Results:**

In the successive bilateral SSNHL group, older average onset age (48.67 ± 15.36 vs. 42.71 ± 13.58, *p* < 0.05), higher male to female ratio (18 : 15 vs. 112 : 103, *p* > 0.05), and poorer therapeutic efficacy (12% vs. 59%, *p* < 0.01) were observed than those in the unilateral SSNHL group. Compared to the unilateral SSNHL group, NLR, MLR, and PLR in the successive bilateral SSNHL group were significantly higher (NLR: 5.72 ± 2.23 vs. 4.45 ± 2.82, *p* = 0.01; MLR: 0.25 ± 0.15 vs. 0.17 ± 0.11, *p* < 0.01; PLR: 190.70 ± 69.79 vs. 148.18 ± 65.67; *p* < 0.01); the LDL level was significantly higher; yet, the HDL level was significantly lower (LDL: 3.79 ± 0.53 vs. 3.49 ± 0.74; HDL: 1.33 ± 0.32 vs. 1.44 ± 0.26; *p* < 0.05 for both); fibrinogen was significantly higher (4.03 ± 0.47 vs. 3.70 ± 0.65; *p* < 0.01). Logistic regression analysis demonstrated that the risk factors for successive bilateral SSNHL included age, NLR, MLR, PLR, LDL, HDL, diabetes, and fibrinogen. However, only NLR, MLR, PLR, diabetes, LDL, and HDL independently predicted successive bilateral SSNHL.

**Conclusion:**

Selected blood inflammatory markers combined with metabolic parameters were positively correlated with successive bilateral SSNHL.

## 1. Introduction

Sudden sensorineural hearing loss (SSNHL) was defined as sensorineural hearing loss of at least 30 dB over three consecutive frequencies in less than 3 days. The incidence was rare ranging from 5 to 20 per 100,000 individuals.

In up to 90% of cases, SSNHL was usually presenting idiopathic, and 95% of the SSNHL patients occurred unilaterally [1]. In contrast, bilateral SSNHL was rare, representing around 5% of cases [2, 3]. In comparison with unilateral SSNHL, bilateral SSNHL was drawing wider and wider public attention because of severer hearing loss and poorer prognosis [4]. According to the interval between the currently affected ear and the firstly affected ear, bilateral SSNHL could be generally categorized into two types: simultaneous (within 3 days) or sequential (from 3 to 30 days) [5].

However, Wang et al. and we observed that there was another special and interesting category, in which patients suffered from SSNHL in the currently affected ear more than 1 year postcontralateral ear experienced SSNHL. In this group, the only ear capable of practical hearing appeared to be vulnerable to systemic pathology or living habits, especially when the contralateral ear experienced moderately severe or worse hearing loss. Among them, the response to the standard treatment remained poor, accompanied with further disturbance in verbal communication or quality of life [6].

The exact etiopathogenesis of the SSNHL remained unclear. Microcirculatory failure, prothrombotic susceptibility, and inflammatory state have been mostly hypothesized. It has been reported that NLR, PLR, and MLR predicted the chronic inflammation status in patients with SSNHL [7]. Metabolic disorders were highly related to microcirculation disorders [8]. Prothrombotic susceptibility has been demonstrated to be one of the causes for ischemic changes in the inner ear [9]. Yet, little was known about the role of high risks in the prediction of successive bilateral SSNHL.

Therefore, we designed this study to explore whether peripheral blood inflammatory markers combined with metabolic or hemostatic parameters could predict incidence of successive bilateral SSNHL.

## 2. Patients and Methods

### 2.1. Patient Population

We retrospectively reviewed 248 medical records of patients with successive bilateral or unilateral SSNHL admitted in our hospital between 2011 and 2015. The inclusion criteria were as follows: (1) SSNHL of more than 30 dB appearing on at least three consecutive frequencies within 3 days; (2) for successive bilateral SSNHL, bilateral ears were attacked successively with a certain interval (≥1 year), not simultaneously or sequentially; (3) for unilateral SSNHL, unilateral ear was attacked; (4) age ≥ 14 years; (5) admitted in hospital within 7 days from onset; (6) followed up until their hearing were fixed; (7) negative MRI cranial nerve VIII pathology findings; and (8) absence of neurologic disorder, head injury, history of otologic operation, drug-related ototoxicity, noise-induced hearing loss, or Meniere disease. Medical records were also reviewed for gender, age at the onset of current hearing loss, past medical history (including hypertension and diabetes), pharmacological history of statins, antihypertensive therapy (including nitrates, angiotensin-converting enzyme inhibitors, beta blockers, angiotensin receptor blockers, and calcium channel blockers), and personal history (including smoking and alcohol consumption).

### 2.2. Ethical Considerations

The study protocol was in compliance with the Code of Ethics of the World Medical Association (Declaration of Helsinki) and approved by the Institutional Review Board at Sun Yat-sen Memorial Hospital, Sun Yat-sen University. Informed consent was obtained from all patients.

### 2.3. Hematologic Examinations

Preoperative blood samples were routinely collected within 7 days after onset of hearing loss and before treatment. Clinical chemistry determinations were made for glucose, total cholesterol, high-density lipoprotein (HDL, mmol/L), low-density lipoprotein (LDL, mmol/L), and serum triglyceride levels (mmol/L). Hematology determinations were made for neutrophil count, lymphocyte count, monocyte count, and platelet count. Neutrophil lymphocyte ratio (NLR), platelet lymphocyte ratio (PLR), and monocyte lymphocyte ratio (MLR) were defined as the ratio of neutrophil, platelet, and monocyte to lymphocyte, respectively. Hemostatic determinations were made for prothrombin time (PT, sec), activated partial thromboplastin time (APTT, sec), and fibrinogen (g/L).

### 2.4. Treatment Process

Patients were admitted to hospital and treated with a 14-day standard therapeutic protocol. In this paradigm, adults started treatment with 1 mg/kg/d oral prednisolone for the first 4 days (60 mg, maximum), with this dosage gradually tapered by 10 mg every 2 days for the following 10 days.

### 2.5. Audiometric Evaluation

Pure-tone audiometry was evaluated before and 30 days posttreatment. Pure-tone average (PTA) was calculated as the average of thresholds (dB HL) at four frequencies of 500, 1000, 2000, and 4000 Hz. Based on audiogram, severity of hearing loss was graded by PTA as mild (26–40 dB), moderate (41–55 dB), moderately severe (56–70 dB), severe (71–90 dB), or profound (>91 dB). Treatment outcomes was evaluated by PTA as follows: (1) complete recovery, defined as the final hearing level ≤ 25 dB, or follow-up PTA returning to within 10 dB of the pre-SSNHL levels or the unaffected ear; (2) partial recovery, defined as hearing gain ≥ 15 dB, or follow-up PTA returning to within 50% of the pre-SSNHL level or the unaffected ear; and (3) no recovery, defined as hearing gain < 15 dB, or follow-up PTA returning to less than 50% of the pre-SSNHL levels or the unaffected ear.

### 2.6. Statistical Analysis

All quantitative data were described as mean ± SD. Categorical data were presented as frequencies or proportion. Independent variables were assumed to be fixed to normal distribution and equal variances by the Kolmogorov-Smirnov test and homogeneity of the variance test, respectively. Between two groups, basic comparative statistics for quantitative variables were performed using Student's two-tail *t* test. If independent variables were not fixed to normal distribution and homogeneity of variance, Mann-Whitney *U* test was applied. The chi-square test was applied for categorical data. Receiver operating characteristic (ROC) curves were plotted to determine the optimum cutoff points. The area under curve (AUC) was used as an estimation of diagnostic accuracy. Influence of hematological parameters and various clinical variables on the prediction of successive bilateral SSNHL was assessed using univariate and multivariate binary logistic regression analysis. Fisher's exact test was adopted to test the comparison of effective rate between two groups. Statistical analysis was performed by using SPSS 21.0 software (SPSS, Chicago, IL). A value of *p* < 0.05 was considered significant.

## 3. Results

### 3.1. Patient Characteristics

**Figure 1:**
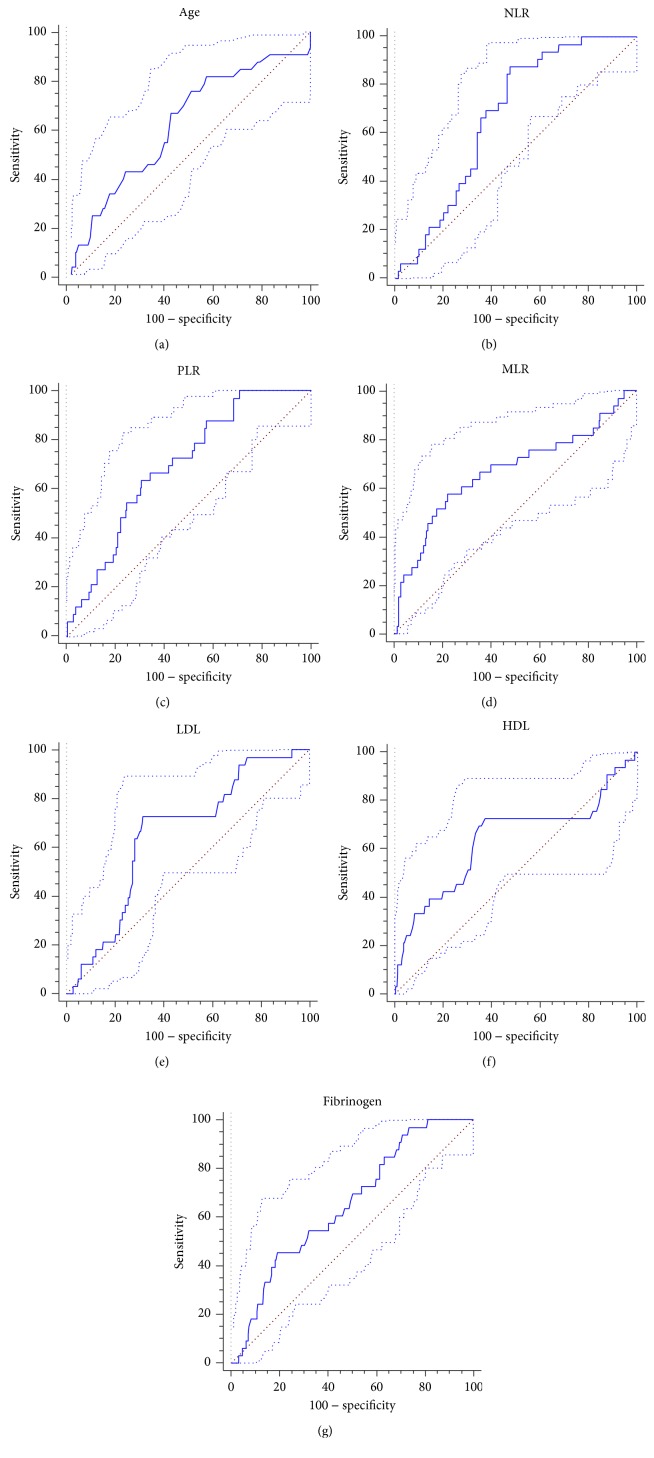
ROC curve for age, NLR, PLR, MLR, LDL, HDL, and fibrinogen. (a) Age (AUC, 0.6262; 95% CI, 0.562-0.686; *p* < 0.05); (b) NLR (AUC, 0.673; 95% CI, 0.611-0.731; *p* < 0.01); (c) PLR (AUC, 0.648; 95% CI, 0.585-0.707; *p* < 0.01); (d) MLR (AUC, 0.670; 95% CI, 0.607-0.728; *p* < 0.01); (e) LDL (AUC, 0.654; 95% CI, 0.591-0.714; *p* < 0.01); (f) HDL (AUC, 0.637; 95% CI, 0.574-0.697; *p* < 0.05); (g) fibrinogen (AUC, 0.651; 95% CI, 0.588-0.711; *p* < 0.01). ROC: receiver operating characteristic; AUC: area under curve; CI: confidence interval; NLR: neutrophil lymphocyte ratio; PLR: platelet lymphocyte ratio; MLR: monocyte lymphocyte ratio; LDL: low-density lipoprotein; HDL: high-density lipoprotein.

The study cohort comprised 248 patients, including 130 (52%) men and 118 (48%) women. Patient characteristics based on SSNHL category are presented in Table 1. In our cohort, 33 patients were diagnosed of successive bilateral SSNHL and 215 were diagnosed of unilateral SSNHL. The mean age at diagnosis was 48.67 ± 15.36 years in the successive bilateral SSNHL group and 42.71 ± 13.58 years in the unilateral SSNHL group (*p* < 0.05). Therefore, an ROC curve for successive bilateral SSNHL was plotted to determine the optimum cutoff values for age, which was presenting as >42 (age: AUC, 0.626; 95% confidence interval (CI), 0.562-0.686; *p* < 0.05) (Figure 1(a)). The gender portion (male to female ratio) was 18 : 15 in the successive bilateral SSNHL group and 112 : 103 in the unilateral SSNHL group (*p* > 0.05). The two groups were similar in proportion of smoking (36% vs. 31%), alcohol consumption (15% vs. 18%), statins (24% vs. 21%), antihypertensive therapy (21% vs. 19%), and accompanying symptoms such as dizziness (36% vs. 33%), tinnitus (70% vs. 68%), and ear fullness (55% vs. 52%) (*p* > 0.05 for all) (Table 1).

### 3.2. Comparison of Inflammatory Parameters between Two Groups

**Figure 2:**
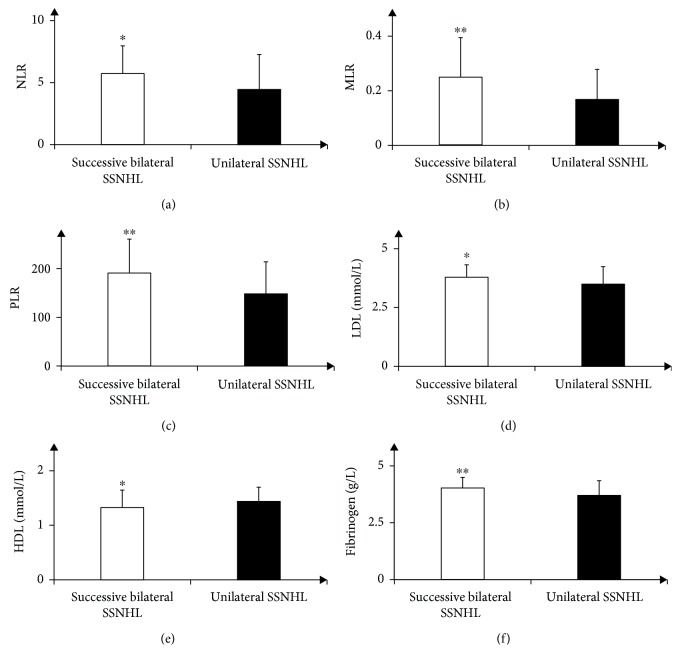
Comparison of peripheral inflammation markers (NLR, PLR, and MLR), metabolic parameters (LDL and HDL), and fibrinogen between two groups. (a) Comparison of NLR between successive bilateral SSNHL and unilateral SSNHL (5.72 ± 2.23 vs. 4.45 ± 2.82, *p* < 0.05); (b) comparison of PLR between successive bilateral SSNHL and unilateral SSNHL (190.70 ± 69.79 vs. 148.18 ± 65.67; *p* < 0.01); (c) comparison of MLR between successive bilateral SSNHL and unilateral SSNHL (0.25 ± 0.15 vs. 0.17 ± 0.11, *p* < 0.01); (d) comparison of LDL between successive bilateral SSNHL and unilateral SSNHL (3.79 ± 0.53 vs. 3.49 ± 0.74, *p* < 0.05); (e) comparison of HDL between successive bilateral SSNHL and unilateral SSNHL (1.33 ± 0.32 vs. 1.44 ± 0.26, *p* < 0.05); (f) comparison of fibrinogen between successive bilateral SSNHL and unilateral SSNHL (4.03 ± 0.47 vs. 3.70 ± 0.65; *p* < 0.01); ^∗^*p* < 0.05, ^∗∗^*p* < 0.01, vs. unilateral SSNHL; NLR: neutrophil lymphocyte ratio; PLR: platelet lymphocyte ratio; MLR: monocyte lymphocyte ratio; LDL: low-density lipoprotein; HDL: high-density lipoprotein; SSNHL: sudden sensorineural hearing loss.

Significant differences were found in NLR, MLR, and PLR between two groups. Mean NLR, MLR, and PLR values were significantly higher in the bilateral successive SSNHL group when compared to the unilateral SSNHL group (NLR: 5.72 ± 2.23 vs. 4.45 ± 2.82, *p* < 0.05; MLR: 0.25 ± 0.15 vs 0.17 ± 0.11, *p* < 0.01; PLR: 190.70 ± 69.79 vs. 148.18 ± 65.67; *p* < 0.01) (Table 1 and Figures 2(a)–2(c)). Therefore, an ROC curve for successive bilateral SSNHL was plotted to determine the optimum cutoff values for NLR (>3.91), MLR (>0.24), and PLR (>166.59) (NLR: AUC, 0.673; 95% CI, 0.611-0.731; MLR: AUC, 0.670; 95% CI, 0.607-0.728; PLR: AUC, 0.648; 95% CI, 0.585-0.707; *p* < 0.01 for all) (Figures 1(b)–1(d)).

### 3.3. Comparison of Metabolic Parameters between Two Groups

Significant differences were found in HDL and LDL between two groups. When compared with the unilateral SSNHL group, mean LDL values were significantly higher; yet, mean HDL values were significantly lower in the bilateral successive SSNHL group (LDL: 3.79 ± 0.53 vs. 3.49 ± 0.74; HDL: 1.33 ± 0.32 vs. 1.44 ± 0.26; *p* < 0.05 for both) (Table 1 and Figures 2(d) and 2(e)). Therefore, an ROC curve for successive bilateral SSNHL was plotted to determine the optimum cutoff values for LDL (>3.52) and HDL (<1.42) (LDL: AUC, 0.654; 95% CI, 0.591-0.714, *p* < 0.01; HDL: AUC, 0.637; 95% CI, 0.574-0.697, *p* < 0.05) (Figures 1(e) and 1(f)). Besides, the proportion of diabetes in the bilateral successive SSNHL group was significantly higher than the unilateral SSNHL group (57% vs. 32%, *p* < 0.01) (Table 1). No significant differences were found in the proportion of hypertension (24% vs. 20%), total cholesterol (5.74 ± 0.63 vs. 5.78 ± 3.16), and triglyceride (1.47 ± 0.56 vs. 1.40 ± 1.23) (*p* > 0.05 for all) (Table 1).

### 3.4. Comparison of Hemostatic Parameters between Two Groups

Significant differences were found in fibrinogen between two groups. Mean fibrinogen values were significantly higher in the bilateral successive SSNHL group compared with the unilateral SSNHL group (4.03 ± 0.47 vs. 3.70 ± 0.65; *p* < 0.01) (Table 1 and Figure 2(f)). Therefore, an ROC curve for successive bilateral SSNHL was plotted to determine the optimum cutoff values for fibrinogen (>4.25) (AUC, 0.651; 95% CI, 0.588-0.711, *p* < 0.01) (Figure 1(g)). No significant differences were found in PT and APTT (PT: 10.94 ± 0.82 vs. 11.51 ± 0.63; APTT: 25.94 ± 2.55 vs. 26.44 ± 3.51; *p* > 0.05 for both) (Table 1).

### 3.5. Impact of Inflammatory, Hemostatic, and Metabolic Parameters and Other Clinical Variables

Factors related to the pathogenesis of successive bilateral SSNHL were evaluated using logistic regression analysis. In univariate analysis, age > 42 years (odds ratio (OR), 0.332; 95% CI, 0.148-0.748; *p* < 0.01), NLR > 3.91 (OR, 0.129; 95% CI, 0.044-0.380; *p* < 0.01), MLR > 0.24 (OR, 0.212; 95% CI, 0.099-0.453, *p* < 0.01), PLR > 166.59 (OR, 0.259; 95% CI, 0.120-0.556; *p* < 0.01), LDL > 3.52 (OR, 0.177; 95% CI, 0.078-0.402; *p* < 0.01), HDL < 1.42 (OR, 4.122; 95% CI, 1.865-9.110; *p* < 0.01), diabetes (OR, 0.334; 95% CI, 0.158-0.705; *p* < 0.01), and fibrinogen > 4.25 (OR, 0.318; 95% CI, 0.149-0.679; *p* < 0.01) were identified as risk factors for successive bilateral SSNHL, while no significant differences were observed in gender, hypertension, smoking, alcohol consumption, statins, and antihypertensive therapy (Table 2). However, on multivariate analysis, only NLR (OR, 0.429; 95% CI, 0.115-1.596; *p* < 0.05), MLR (OR, 0.134; 95% CI, 0.045-0.399; *p* < 0.01), PLR (OR, 0.348; 95% CI, 0.122-0.997; *p* < 0.05), diabetes (OR, 0.245; 95% CI, 0.082-0.730; *p* < 0.05), LDL (OR, 0.216; 95% CI, 0.081-0.579; *p* < 0.01), and HDL (OR, 4.423; 95% CI, 1.624-12.048; *p* < 0.01) were regarded as independent prognostic factors (Table 2).

### 3.6. Comparison of Treatment Outcomes between Two Groups

In the successive bilateral SSNHL group, 1 case exhibited complete recovery in the currently affected ear and 3 cases exhibited partial recovery, with the total effective rate (complete and partial recovery) of 12%. In the unilateral SSNHL group, the total effective rate reached 59%, which was significantly higher than the successive bilateral group (*p* < 0.01) (Table 1).

## 4. Discussion

SSNHL was a common otologic emergency, with unilateral SSNHL accounting for nearly 95% [2]. Compared to unilateral SSNHL, bilateral SSNHL was presenting with much poorer prognosis [10, 11]. Except for simultaneous or sequential bilateral SSNHL, it was reported there was another special bilateral SSNHL. This was defined as successive bilateral SSNHL, in which patients were more vulnerable to suffer from more severe hearing impairment and the treatment outcomes were much poorer [6]. Regarding the completely different profile, it raised the necessity to investigate the potential risk factors related to bilateral successive SSNHL. The exact etiopathogenesis of the SSNHL remained unclear. Microcirculatory failure, prothrombotic susceptibility, and inflammatory state were mostly hypothesized. Our study proved that selected blood inflammatory combined with metabolic parameters better predicted the pathogenesis of successive bilateral SSNHL.

In our study, the average age of successive bilateral SSNHL patients was 48.67 ± 15.36 years, which was significantly older than the unilateral SSNHL patients (42.71 ± 13.58 years). Our results were consistent with previous studies. In Sara et al.'s study, the mean age of onset for unilateral SSNHL was 41 years [12], while Fetterman et al. reported a mean age of 51 years for bilateral SSNHL [5]. It was suggested that more attention should be paid on the older patients, and we speculated that it was because older patients were more vulnerable to metabolic disorders or cardiovascular disorders [13].

Recently, the causes of SSNHL were mostly focused on chronic inflammation [7]. It was hypothesized that systemic stress-related chronic inflammation would impair endothelial function and then produce atherosclerosis, therefore finally leading to ischemic changes in microvascular structures. As an outward manifestation of inflammatory state, peripheral blood NLR, MLR, and PLR might serve as a convenient, reliable, and cost-effective indicator for the pathogenesis of SSNHL. NLR and MLR have been defined as a novel potential marker to determine the level of inflammation. Besides, an elevated PLR might therefore lead to an increase in vascular endpoints such as atherosclerosis [14, 15]. Previous studies demonstrated that for SSNHL, the optimal cutoff values for NLR and PLR was >4.48 and >146.75, respectively [14]. In our study, we discovered that NLR > 3.91 and PLR > 166.59 were reliable independent risk factors to predict the pathogenesis of successive bilateral SSNHL. Comparably, our results were consistent with the aforementioned criteria. However, little was known about the role of MLR in predicting SSNHL; our study has made up for this blank field, showing that MLR > 0.24 better predicted successive bilateral SSNHL.

As we know, metabolic syndrome, like dyslipidemia, hypertension, and diabetes, was highly related to microangiopathy. Dyslipidemia was characterized by low HDL, high LDL, hypercholesterolemia, and/or triglyceridemia. It would disrupt blood supply by plaque formation, vascular remodeling, endothelial dysfunction, vascular inflammation, and vessel obstruction. Meanwhile, the cochlea was a highly metabolic organ, which was supplied by the labyrinthine artery without collateral arterial blood flow. This mechanism made the cochlea vulnerable to microvascular ischemia [8]. Ciccone et al. have confirmed that the ISSHL patients were at a higher cardiovascular risk, which suggests the vascular genesis of cochlear damage. Moreover, asymmetrical alterations in venous extracranial hemodynamics could contribute to the pathogenesis of SSNHL [16]. Besides, they first mentioned that hearing thresholds of the contralateral ear were related to carotid intima-media thickness and flow-mediated dilation of the brachial artery representing subclinical atherosclerosis [13]. Our data supported that patients with higher LDL and lower HDL values were strongly associated with successive bilateral SSNHL, with the optimal cutoff values of >3.52 mmol/L and <1.42 mmol/L for LDL and HDL, respectively. Besides, it has been proven that correction of dyslipidemia in patients with chronic phase SSNHL was found to improve hearing, providing further evidence that dyslipidemia was associated with the onset of impaired hearing and its prognosis [8]. Hereby, we strongly speculated that dyslipidemia participated in the development of sudden deafness, and it was better to monitor the LDL or HDL level after unilateral SSNHL, and correction of dyslipidemia would avoid the future occurrence of successive bilateral SSNHL. Moreover, hypertension and diabetes were previously reported as risk factors for SSNHL. However, in our study, there were significant differences only in the proportion of diabetes but not in hypertension. Many studies suggested that diabetes were more likely to affect the internal environment, which would interfere the development of sudden deafness; however, hypertension was reported to partially restrict the reaction to therapy or recovery [17].

As to prothrombotic susceptibility, we explored the role of a series of prothrombotic parameters including APTT, PT, and fibrinogen in the prediction of successive bilateral SSNHL. Significant differences were only observed in fibrinogen but not in PT and APTT. Our data demonstrated that patients with higher fibrinogen value were strongly associated with successive bilateral SSNHL, with the optimal cutoff values of >4.25 g/L for fibrinogen. This was consistent with previous studies. It has been demonstrated that high fibrinogen levels might indicate ischemic changes in the inner ear. It was related to high blood viscosity and thus decreased blood supply to affected areas [9]. That was also the reason why Canis et al. recommended fibrinogen apheresis in patients presenting with high fibrinogen levels [18].

At last, the proportion of smokers among patients in the successive bilateral SSNHL group was not significantly higher compared to the unilateral SSNHL. This was consistent with previous finding that there was no direct association between smoking and onset of sudden deafness. It was speculated that smoking would contribute to poorer response to traditional therapy rather than participate in the pathogenesis. Nakamura et al. have clarified that therapeutic outcomes in smokers with SSNHL were poorer than nonsmokers with SSNHL, accompanied with higher recurrence rates [19, 20].

There were several limitations in our study: (1) the patients were enrolled from one single institution, and the data were analyzed retrospectively; (2) the prognostic role of inflammatory markers associated with metabolic features needed to be validated in future prospectively designed investigations; (3) a blank control group would be better included in our future prospective studies.

## 5. Conclusion

The overall therapeutic efficacy for successive bilateral SSNHL was significantly poorer than unilateral SSNHL. We found that peripheral inflammation markers (including NLR, PLR, and MLR) combined with metabolic parameters (including LDL, HDL, and diabetes) were significant independent prediction factors for successive bilateral SSNHL, therefore providing more accurate information for further prophylaxis.

## Figures and Tables

**Table 1:** Patient characteristics based on SSNHL type.

Parameters	Successive bilateral SSNHL (*n* = 33)	Unilateral SSNHL (*n* = 215)
Age (years), mean ± SD	48.67 ± 15.36^∗^	42.71 ± 13.58
Age ≤ 42 years:>42 years, number	9 : 24	114 : 101
Males : females, number	18 : 15	112 : 103
NLR, mean ± SD	5.72 ± 2.23^∗^	4.45 ± 2.82
NLR ≤ 3.91:>3.91, number	4 : 29	111 : 104
MLR, mean ± SD	0.25±0.15^∗∗^	0.17 ± 0.11
MLR ≤ 0.24:>0.24, number	14 : 19	167 : 48
PLR, mean ± SD	190.70±69.79^∗∗^	148.18 ± 65.67
PLR ≤ 166.59:>166.59, number	12 : 21	148 : 67
LDL (mmol/L), mean ± SD	3.79 ± 0.53^∗^	3.49 ± 0.74
LDL ≤ 3.52:>3.52, number	9 : 24	146 : 69
HDL (mmol/L), mean ± SD	1.33 ± 0.32^∗^	1.44 ± 0.26
HDL ≤ 1.42:>1.42, number	23 : 10	77 : 138
Total cholesterol (mmol/L), mean ± SD	5.74 ± 0.63	5.78 ± 3.16
Triglyceride (mmol/L), mean ± SD	1.47 ± 0.56	1.40 ± 1.23
Fibrinogen (g/L), mean ± SD	4.03±0.47^∗∗^	3.70 ± 0.65
Fibrinogen ≤ 4.25:>4.25, number	18 : 15	170 : 45
PT (sec), mean ± SD	10.94 ± 0.82	11.51 ± 0.63
APTT (sec), mean ± SD	25.94 ± 2.55	26.44 ± 3.51
Smoking, number (%)	12 (36%)	66 (31%)
Alcohol consumption, number (%)	5 (15%)	38 (18%)
Hypertension, number (%)	8 (24%)	42 (20%)
Diabetes, number (%)	19 (57%)^∗∗^	67 (32%)
Statins, number (%)	8 (24%)	45 (21%)
Antihypertensive therapy, number (%)	7 (21%)	40 (19%)
Nitrates	0 (0%)	0 (0%)
Angiotensin-converting enzyme inhibitors	2 (6%)	10 (5%)
Beta blockers	1 (3%)	5 (2%)
Angiotensin receptor blockers	2 (6%)	10 (5%)
Calcium channel blockers	2 (6%)	15 (7%)
Accompanying symptoms, number (%)		
Dizziness	12 (36%)	71 (33%)
Tinnitus	23 (70%)	146 (68%)
Ear fullness	18 (55%)	112 (52%)
Total effective rate, number (%)	4 (12%)^∗∗^	126 (59%)

^∗^ *p* < 0.05, ^∗∗^*p* < 0.01, vs. unilateral SSNHL; SSNHL: sudden sensorineural hearing loss; NLR: neutrophil lymphocyte ratio; PLR: platelet lymphocyte ratio; MLR: monocyte lymphocyte ratio; LDL: low-density lipoprotein; HDL: high-density lipoprotein; PT: prothrombin time; APTT: activated partial thromboplastin time.

**Table 2:** Univariate and multivariate logistic regression analysis of prediction for successive bilateral SSNHL in patients with SSNHL (*n* = 248).

Characteristics	Univariate			Multivariate		
	*p* value	OR	95% CI	*p* value	OR	95% CI
Age > 42 years	**<0.01**	0.332	0.148-0.748	0.809	0.882	0.319-2.440
Gender (male)	>0.05	0.544	0.251-1.176			
NLR (>3.91)	**<0.01**	0.129	0.044-0.380	**<0.05**	0.429	0.115-1.596
PLR (>166.59)	**<0.01**	0.259	0.120-0.556	**<0.01**	0.348	0.122-0.997
MLR (>0.24)	**<0.01**	0.212	0.099-0.453	**<0.01**	0.134	0.045-0.399
LDL (>3.52)	**<0.01**	0.177	0.078-0.402	**<0.01**	0.216	0.081-0.579
HDL (<1.42)	**<0.01**	4.122	1.865-9.110	**<0.01**	4.423	1.624-12.048
Fibrinogen (>4.25)	**<0.01**	0.318	0.149-0.679	>0.05	0.561	0.214-1.468
Diabetes	**<0.01**	0.334	0.158-0.705	**<0.05**	0.245	0.082-0.730
Hypertension	>0.05	0.759	0.320-1.801			
Smoking	>0.05	0.775	0.360-1.668			
Alcohol consumption	>0.05	1.202	0.436-3.314			
Statins	>0.05	0.827	0.350-1.957			
Antihypertensive therapy	>0.05	0.849	0.344-2.093			

SSNHL: sudden sensorineural hearing loss; OR: odds ratio; CI: confidential interval; NLR: neutrophil lymphocyte ratio; PLR: platelet lymphocyte ratio; MLR: monocyte lymphocyte ratio; LDL: low-density lipoprotein; HDL: high-density lipoprotein.

## Data Availability

The data used to support the findings of this study are available from the corresponding author upon request.
